# Supplementary material for: Effects of Environmental Enrichment on Exposure to Human-Relevant Mixtures of Endocrine Disrupting Chemicals in Zebrafish
Source: Animals (Basel). 2024 Apr 25;14(9):1296. doi: 10.3390/ani14091296 (PMC11083384; doi:10.3390/ani14091296)
Supplement: Supplementary file 1 [file animals-14-01296-s001.zip › animals-2848301-supplementary.pdf]

# Supplementary materials

Supplementary materials for "*Effects of environmental enrichment on exposure to human-relevant mixtures of endocrine disrupting chemicals in zebrafish*". Authors: Lina Birgersson, Sanne Odenlund and Joachim Sturve

**Supplementary Table S1.** Composition and concentration of EDC mixtures associated with low birth weight within the EDC-MixRisk project.

| Mixture component (parent compound) | Name | MIX G0 | MIX G1 |
|---|---|---|---|
| MEP (DEP) | Mono-ethyl phthalate | 2.34E-08 | 3.204E-08 |
| MBP (DBP) | Mono-butyl phthalate | 2.00E-08 | 2.855E-08 |
| MBzP (BBzP) | Mono-benzyl phthalate | 9.1E-09 | 5.68E-09 |
| MINP (DINP) | Mono-isononyl phthalate | 1.79E-08 | - |
| MEHP (DEHP) | Mono-ethyl hexyl phthalate | 1.24E-08 | 2.051E-08 |
| DPP | Dipentyl phthalate | - | 4.9E-10 |
| TCS | Triclosan | 2.6E-09 | 3,00E-10 |
| PFOS | Perfluorooctane sulfonate | 8.9E-09 | 1.048E-08 |
| PFOA | Perfluorooctanoic acid | 2.9E-09 | 3.89E-09 |
| PFHxS | Perfluorohexane sulfonate | 2.8E-09 | 3.28E-09 |
| 3-PBA | 3-Phenoxybenzoic acid | - | 1.1E-10 |
| p,p′DDE | p,p'-dichlorodiphenyldichloroethylene | - | 5.9E-10 |
| HCB | Hexachlorobenzene | - | 1.6E-10 |
| MINCH (DINCH) | 1,2-Cyclohexane dicarboxylic acid diisononyl ester | - | 5.2E-10 |
| 2-OH-PH | 2-hydroxyphenanthrene | - | 1.36E-09 |

**Supplementary Table S2.** List of genes tested and primers used for RT-qPCR

| Gene | Forward primer (5'-3') | Reverse primer (5'-3') | Gene Accession |
|---|---|---|---|
| *dio1* | GTTCAAACAGCTTGTCAAGGACT | AGCAAGCCTCTCCTCCAAGTT | NM_001324404.1 |
| *dio2* | GCATAGGCAGTCGCTCATTT | TGTGGTCTCTCATCCAACCA | NM_212789.4 |
| *dio3* | GCGCGTACGGAGCTTACTTC | AGCTCGGAGATGCGGAATCC | NM_001256003.1 |
| *thra* | CTATGAACAGCACATCCGACAAGAG | CACACCACACACGGCTCATC | NM_131396.1 |
| *thrb* | TGGGAGATGATACGGGTTGT | ATAGGTGCCGATCCAATGTC | NM_131340.1 |
| *actβ* | GCAGAAGGAGATCACATCCCTGGC | CATTGCCGTCACCTTCACCGTTC | NM_181601.4 |
| *gapdh* | TGACCTGATGGCACACATGG | GATGGGAGAATGGTCGCGTA | NM_001115114.1 |
| *rplp0* | CATCTCGCCCTTCTCCTACG | AGGAATCTCTTGTGCAGGGC | NM_131580.2 |
